# Presumptive Acute Neural Toxoplasmosis in a Captive Red-Necked Wallaby (*Macropus rufogriseus*)

**DOI:** 10.4061/2010/561212

**Published:** 2010-06-17

**Authors:** Carlos Hermosilla, Nikola Pantchev, Nicole Gies, Anja Taubert

**Affiliations:** ^1^Department of Pathology and Infectious Diseases, Royal Veterinary College, AL 97 TA Hertfordshire, UK; ^2^Vet Med Labor GmbH IDEXX Laboratories, 71638 Ludwigsburg, Germany; ^3^Veterinary Clinic, Weingarten 2, 59065 Hamm, Germany; ^4^Institute of Parasitology, Justus Liebig University Giessen, 35392 Giessen, Germany

## Abstract

A red-necked male wallaby (*Macropus rufogriseus*) from a German zoo was presented for acute onset of severe neurological signs, including head tremor, lethargy, unresponsiveness, and weakness. Serum biochemical abnormalities included increased LDH- and AST-levels, hyperproteinaemia, and reduced ALT-, ALP-, and creatinine-levels. The wallaby was found serologically positive for *Toxoplasma gondii* by the indirect haemagglutination test. After initiation of therapy by subcutaneous injections of trimethoprim/sulfadoxin, amelioration of neurological signs was noted and after 10 days the affected wallaby recovered. *T. gondii* can be confirmed rapidly by serology, and immediate therapy may reduce clinical illness and fatality of the disease within captive macropods.

*Toxoplasma gondii *infections underlie complex immunological regulations, and clinical disease is generally limited to naive, immature, or immunosuppressed animals [1]. However, in adult Australian marsupials, particularly macropods (Macropodidae), toxoplasmosis can cause high morbidity and mortality [2]. Wallabies are reported as the most susceptible in this family [3]. Fatal generalized toxoplasmosis in red-necked wallabies (*Macropus rufogriseus*) has been reported recently [4, 5]. In this report, we describe a case of presumptive acute toxoplasmosis with a history of neurological signs, in a captive red-necked wallaby (*Macropus rufogriseus*) from the zoo of Hamm. 

In September 2007, an 8.5-year-old red-necked male wallaby (*Macropus rufogriseus*) displaying severe neurological signs, including head tremor, lethargy, unresponsiveness, and weakness (see Figure 1) was presented to the veterinary surgeon of the Zoo of Hamm, (59065, Germany). Physical examination revealed pyrexia (39°C), neurological and ophthalmic signs including nystagmus and bilateral epiphora. Serum biochemical abnormalities included increased LDH- and AST-levels, hyperproteinaemia, and reduced ALT-, ALP-, and creatinine-levels. Despite immediate supportive therapy with antibiotics (enrofloxacin, 10 mg/kg body weight, s.c., Baytril, Bayer) and corticosteroids (dexamethasone, 0.1 mg/kg body weight, s.c., Medistar-Dexamethason, Medistar), there was no improvement. After one week of stagnation, a tentative diagnosis of toxoplasmosis was made. For confirmation, blood samples were taken for serological analysis. The wallaby was found serologically positive for *T. gondii*, showing high antibody titres (≥1 : 1024) in the indirect haemagglutination (IHA) test (Cellognost-Toxoplasmosis H, Dade Behring). Neither *T. gondii- *nor *Neospora caninum-*DNA was detected in the EDTA-blood samples by specific PCR [6].

After confirmation of toxoplasmosis, appropriate treatment of the wallaby was initiated. Subcutaneous injections of trimethoprim/sulfadoxin (15 mg/kg body weight daily for 10 days, Sulphix, Bela-Pharm) were administered. Three days after initiation of therapy, amelioration of neurological signs was noted in the wallaby and after 7 days the majority of the clinical signs were no longer apparent. As the infected animal shared premises with two other wallabies and *T. gondii *infection of these could not be excluded, respective blood samples were analysed serologically as well. Both wallabies, which showed no clinical signs of disease, also had high antibody titres (≥1 : 1024) against *T. gondii*. 

Toxoplasmosis has been reported in captive macropods from various zoos worldwide [5, 7]. As red-necked wallabies (*Macropus rufogriseus*) are herbivores, they presumably acquire *T. gondii *by the oral uptake of sporulated oocysts shed from stray cats or other felids [7]. Transplacental and transmammary infections are also reported in joeys [3]. In red-necked wallabies, generalized *T. gondii *infections may be associated with nonsuppurative meningoencephalitis, hepatitis, myositis, myocarditis, keratitis, uveitis, choroidoretinitis, endophthalmitis, or severe enteritis [5]. As such, a broad range of clinical signs, such as depression, lethargy, ataxia, cataracts, unresponsiveness, weakness, diarrhoea, and weight loss have been described. The neurological findings seen in this report, such as depression, nystagmus, and head tremor may reflect an acute meningoencephalitis, bilateral epiphora, and ophthalmic disorders. Serum biochemical abnormalities detected in the sick wallaby, such as hyperproteinaemia, could also indicate an inflammatory process. Increased LDH-levels are often seen in myositis/myocarditis, which have been reported in acute toxoplasmosis in wallabies [5]. Subclinical *T. gondii *infections, as reported in the other species, have also been documented in macropods [8, 9]. Acute toxoplasmosis in this wallaby might have been triggered by individual additional factors, for example, stress. The wallaby mentioned in this report most probably became infected by the ingestion of sporulated oocysts of *T. gondii* spread on the ground of the premises. Interestingly, the premises flanked a local allotment garden area with a stable stray cat population. For many years stray cats have been observed at the Zoo of Hamm and, as in other zoos, they are suspected to be the most likely source of *T. gondii-*oocysts contamination [7].

The case reported here indicates that toxoplasmosis should be considered as a differential diagnosis in cases of neurological disorders in wallabies. *T. gondii* infection can be confirmed rapidly by serology, and immediate therapy may reduce clinical illness and fatality of the disease within captive macropods. The treatment with trimethoprim/sulfonamide has been described to be effective against *T. gondii* and may be used prophylactically in macropods during toxoplasmosis outbreaks [10]. Additionally, the successful treatment with atovaquone in wallabies suffering from ocular toxoplasmosis has recently been described [11].

## Figures and Tables

**Figure 1:**
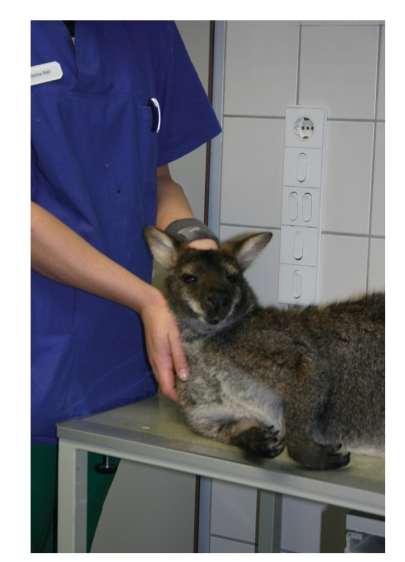
Red-necked wallaby (*Macropus rufogriseus*) showing lethargy and severe weakness as indicated by the inability to keep its head upright.
